# Emergency Care Interventions for Victims of Explosive Ordnance Reduce Mortality: A Modeling Study

**DOI:** 10.1017/S1049023X25101283

**Published:** 2025-08

**Authors:** Hannah B. H. Wild, Benjamin Q. Huynh, Sebastian Kasack, Alex Munyambabazi, Yves Sanou, Yves Nacanabo, Moumini Niaone, Aparna Cheran, Emilie Calvello Hynes, Nicolas Meda, Adam Kushner, Barclay T. Stewart

**Affiliations:** 1. Department of Surgery, University of Seattle, Washington, USA; 2. Explosive Weapons Trauma Care Collective, International Blast Injury Research Network, University of Southampton, Southampton, United Kingdom; 3. Department of Environmental Health and Engineering, Johns Hopkins University, Baltimore, Maryland, USA; 4. Center for Humanitarian Health, Johns Hopkins Bloomberg School of Public Health, Baltimore, Maryland, USA; 5. Mines Advisory Group, Manchester, United Kingdom; 6. Amputee Self Help Network Uganda, Kampala, Uganda; 7. Captain Halassane Coulibaly Military Hospital, Ouagadougou, Burkina Faso; 8. Department of Public Health, University of Joseph Ki-Zerbo, Ouagadougou, Burkina Faso; 9. Department of Social and Behavioral Sciences, Johns Hopkins University, Baltimore, Maryland, USA; 10. World Health Organization, Geneva, Switzerland; 11. Surgeons Overseas, New York, New York, USA; 12. Global Injury Control Section, Harborview Injury Prevention, Washington and Research Center, Seattle, Washington, USA

**Keywords:** blast injury, civilian casualties, conflict, emergency care, emergency care systems, explosive ordnance, explosive weapons, humanitarian mine action, low-resource settings, trauma care

## Abstract

**Background:**

Modern conflicts are characterized by wide-spread use of conventional explosive ordnance (EO), improvised explosive devices (IEDs), and other air-launched explosives. In contrast to advances in military medicine and high-income civilian trauma systems since the United States-led wars in Afghanistan and Iraq, the mortality rate among civilian EO casualties has not decreased in decades. Although humanitarian mine action (HMA) stakeholders have extensive presence and medical capabilities in EO-affected settings, coordination between HMA and health actors has not been leveraged systematically.

**Methods:**

Data from a prior systematic review of emergency care interventions feasible within the context of HMA activities and low-resource health care systems were used to model mortality reduction among EO victims. Interventions were categorized using the World Health Organization (WHO) Emergency Care System Framework sites of “scene,” “transport,” and “facility.” The cumulative impact of the interventions on EO-related mortality was estimated using pooled effect estimates and simulation modeling.

**Results:**

The meta-analysis included 16 reports from 13 countries, representing 127,505 injured persons. Pooled effect estimates across subcategories of emergency care interventions were 0.42 for layperson transportation (95%CI, 0.24-0.74), 0.79 for prehospital notification systems (95%CI, 0.51-1.19), 0.52 for prehospital trauma care training courses (95%CI, 0.46-0.59), 0.67 for facility-based trauma care training courses (95%CI, 0.48-0.92), and 0.66 for facility-based trauma team organization and activation protocols (95%CI, 0.45-0.97). A 68% reduction in mortality (95%UI, 57%-79%) was observed when implementing the full set of interventions in a region with no prior implemented interventions.

**Conclusion:**

Enhanced coordination between HMA and health actors to implement a structured set of emergency care interventions holds potential to significantly reduce preventable death among civilian EO casualties.

## Introduction

Modern armed conflicts are increasingly characterized by the use of explosive weapons, including conventional explosive ordnance (EO), improvised explosive devices (IEDs), and air-launched munitions (eg, barrel bombs, drone-delivered explosives), as well as high-energy explosives including thermobaric weapons.^1,2^ The indiscriminate nature of these weapons has led to a significant shift in the epidemiology of conflict casualties, namely an increasing proportion of non-combatants, women, and children.^3,4^ The use of explosive weapons in populated areas yields particularly devastating effects due to consequences including amplification of primary blast waves in closed environments and destruction of residential infrastructure, utilities, and health facilities.^5^ Beyond the devastation from the wars in Ukraine, Gaza, Syria, and Yemen, the use of explosives causes extensive harm in less-publicized conflicts such as those in Sudan, Myanmar, Tigray, and the Sahel.^6–10^

Humanitarian mine action (HMA) encompasses activities designed to reduce the threat of EO, including survey and clearance, explosive ordnance risk education (EORE), and stockpile destruction.^11^ While HMA stakeholders are not primarily tasked with the provision of health care in EO-affected regions, significant medical, communication, and transport capabilities exist within the HMA sector. For example, each individual on a de-mining team is trained to the level of a basic medic with at least one paramedic-level trained individual in accordance with the International Mine Action Standards (IMAS) 10.40 on Medical Support to De-mining Operations.^12^ Further, certain HMA organizations such as the International Committee of the Red Cross (ICRC; Geneva, Switzerland) and Humanity and Inclusion (HI; Lyon, France) conduct both mine action activities as well as provide emergency, critical, and operative (ECO) care, in addition to on-going rehabilitation services. In 2021, IMAS 13.10 on Victim Assistance was adopted, which delineates provisions and responsibilities towards the assistance for victims of EO, including emergency medical care and transport.^13^ Emergency care systems in low-resource conflict and post-conflict settings are frequently characterized by gaps in infrastructure, materials, and trained personnel. Such gaps create challenges for the implementation of best-guidance practices generated by organizations such as the World Health Organization (WHO; Geneva, Switzerland).

Therefore, HMA holds significant potential to contribute to strengthening emergency care systems in EO-affected settings; however, to date, this potential has not been systematically explored.^14^ At the beginning of the United States-led wars in Afghanistan and Iraq, the case fatality rate among combat casualties was 20%.^15^ Over the course of these wars, iterative improvements to the military Joint Trauma System produced a 44% decrease in mortality among critically injured combat casualties.^15^ In contrast, the case fatality rate among civilian EO casualties remains between 35%-40% and has not decreased in decades.^16,17^ As civilian casualties of EO mount in conflict settings globally, leveraging intersectoral partnerships to reduce preventable death is imperative. In order to define concrete, feasible opportunities for cooperation between HMA actors and emergency care providers, a systematic review of emergency care interventions with relevance to EO casualty care was previously conducted, as well as a qualitative analysis with key informants in both sectors.^18,19^ Building from these reports, the present modeling study was undertaken to estimate the potential mortality reduction of a coordinated set of interventions designed to reduce preventable death among EO casualties in low-resource conflict settings (LRCS).

## Methods

**Figure 1.**
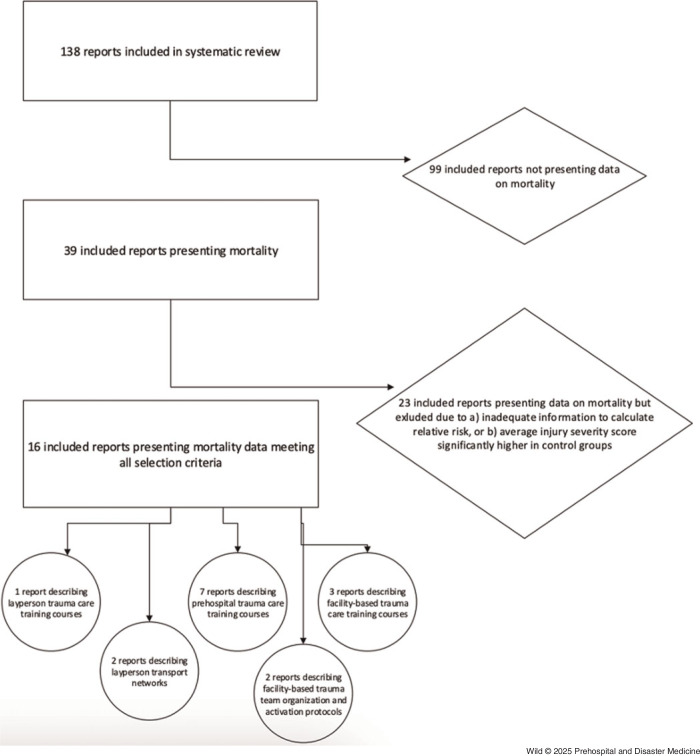
Selection of Included Studies. Note: Eligible reports from prior systematic review - Wild H, Leboa C, Markou-Pappas N, et al (2025).

The approach adopted in this modeling study consisted of selecting reports of relevant emergency care interventions, pooling intervention effect estimates via meta-analyses, and simulation modeling to estimate overall mortality reduction of EO casualties in LRCS (Figure 1).

### Intervention Selection Criteria

Study selection was based on a prior systematic review conducted on emergency care interventions relevant to the care of EO casualties in LRCS.^18^ Studies from the prior systematic review were included in analysis if they:

Focused on emergency care interventions relevant to EO casualty care in LRCS;

Provided point estimates of mortality reduction with uncertainty measurements such as confidence intervals; and

Reported adjusted analyses or ensured comparability of Injury Severity Score (ISS) between treatment and control groups.

Eligible studies were defined as those “relevant to EO casualty care in LRCS.” Therefore, for intervention types where literature specifically on EO casualty care was unavailable, other populations were included (eg, post-partum hemorrhage requiring emergency transport) when such interventions could equally be applied in the context of EO-related injury. Studies with unadjusted analyses were excluded where the mean ISS was significantly higher in the control group compared to the treated group, as this could artificially inflate the observed mortality reduction effect (Figure 1).

As in the prior systematic review, emergency care interventions were categorized into three phases of care corresponding to the (WHO) Emergency Care System Framework “scene,” “transport,” and “facility” schematic: (1) layperson first response (community members without formal medical training); (2) prehospital care (providers with medical training providing care in the prehospital setting); and (3) facility-based care (emergency care by providers in a health facility).^18,20^ Where interventions spanned multiple phases of care (ie, training of both layperson and prehospital personnel), these were classified at the highest level of medical capability to avoid over-estimating the impact of interventions focused on individuals not formally within the health system (ie, community members). The feasibility of integrating emergency care interventions within existing HMA activities was determined through a separate qualitative analysis of key informant interviews with HMA and health stakeholders.^19^ This bundle of interventions was visualized as the Civilian Casualty Care Chain (C-CCC; Supplementary Figure 1 – available online only). Each intervention was then further classified into one of the following subcategories of emergency care and system capacity building: (1) trauma care training courses, (2) transport systems, (3) casualty notification systems, and (4) trauma team organization and activation protocols.^21,22^

For the review process, two study team members independently screened titles, abstracts, and full titles to ensure inclusion criteria were met. Similarly, data extraction from studies was conducted independently by two study team members, with results cross-verified to minimize potential errors.

### Pooling Intervention Effect Estimates

**Figure 2.**
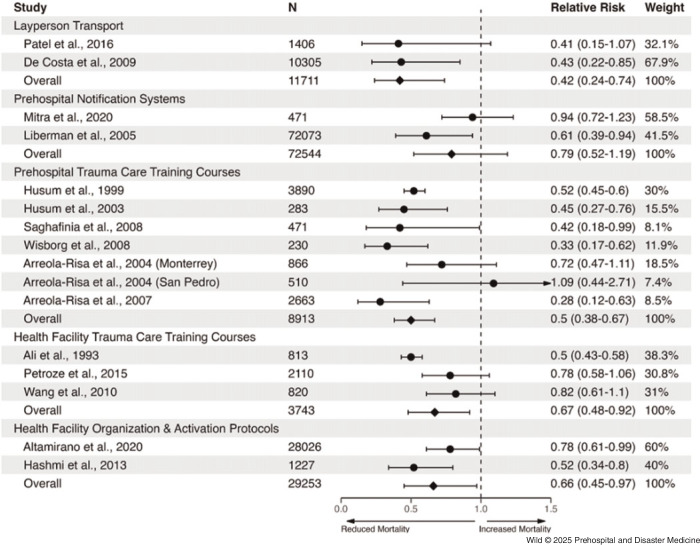
Forest Plots for Meta-Analyses, Stratified by Intervention Category. Note: Circular dots indicate reported relative risk estimates from prior studies. Diamonds, placed in rows denoted as “Overall,” indicate our pooled estimates of relative risk per intervention category via meta-analysis. Uncertainty bars indicate 95% confidence intervals.

For the six intervention subcategories containing more than one report, random effects meta-analyses were conducted to pool effect estimates for each of them (Figure 2; Supplementary Table 1 – available online only). Report results were aggregated based on their reported relative risks and 95% confidence intervals (used to estimate the standard error). Between-study variance was calculated using the Paule-Mandel method and evaluated heterogeneity using I^2^ statistics. For multi-site reports where relative risk estimates were available for each site, results from each site were treated as separate observations for the meta-analysis. For pre-post reports, sample sizes were calculated based only on the pre- and post-intervention periods; for reports where period-specific sample sizes were unavailable, the total sample size was assumed to be split evenly across periods. Leave-one-out meta-analyses were also conducted for intervention subcategories with more than two reports to assess for outliers (Supplementary Figure 2 and Supplementary Figure 3 – available online only).

### Intervention Modeling

The aggregate reduction in mortality was modeled via a Monte Carlo-based simulation with 10,000 iterations. Intervention effects were varied by their estimated variability, assuming a log-normal distribution for each relative risk, defined by mean and standard deviation parameters. Setting the mean as the point estimate for each relative risk, standard deviations were estimated by formulating a constrained optimization problem wherein 95% of values from a lognormal distribution would be inside the 95% confidence intervals from the original relative risk estimates. Formally, the optimization procedure used was as follows:

where *μ*_*rr*_ indicates the point estimate of a relative risk, and *L* and *U* indicate the lower and upper bounds of its 95% confidence interval, respectively. A variety of optimization techniques were used (Nelder-Mead optimization, Newton-like nonlinear minimization, Hooke-Jeeves optimization, Broyden–Fletcher–Goldfarb–Shanno optimization), selecting the result that achieved the lowest objective function value in each case.^23–26^

Mortality reductions from multiple interventions were aggregated by sequentially multiplying relative risks to avoid double counting of mortality reductions, similar to the approach used by the Lives Saved Tool.^27^ To account for the fact that emergency care interventions may target the same physiological mortality pathways (eg, in cases of traumatic hemorrhage, interventions such as lay tourniquet use, early blood transfusion, and timely access to surgical hemorrhage control can all contribute to reducing mortality by addressing similar underlying mechanisms), an interaction factor, *δ*, was introduced that represented diminishing marginal returns in deploying additional interventions.

For example, an aggregate relative risk RR_A_ composed of two mortality reduction interventions would be represented by *RR*_*A*_=(*RR*_1_*(1-(1-*RR*_*2*_)*δ). A further model assumption was imposed that aggregating mortality reduction interventions never increases mortality compared to any individual intervention, setting the end aggregate relative risk to be *RR*_*A*_ *’*= *min*(*RR*_*1*_,*RR*_*2*_,*RR*_*A*_). As the true value of *δ* is not known, this was varied widely, using a uniform distribution between 0.0 and 0.5. As robustness checks, *δ* was fixed at various levels ranging from zero to one, as the original specification assumed a median value of 0.25.

All the analyses were performed with the use of R software, version 4.4.1 (R Project for Statistical Computing; Vienna, Austria). Modeling was performed on a computer with an Apple M3 Max processor with 128 GB of unified memory with macOS version 15.4 (Apple Inc.; Cupertino, California USA).

## Results

### Characteristics of Included Reports

The aforementioned systematic review found 138 reports that described emergency care interventions applicable to civilian casualties from EO in LRCS (Supplementary Figure 1).^18^ Thirty-nine reports presented mortality differences from interventions. Of those 39 reports, 16 met criteria of having sufficient information to calculate relative risks, average ISS scores not significantly higher in control groups, and interventions related to emergency care that could feasibly be implemented within the context of HMA.^28–43^

In the 16 relevant reports, an aggregate population of 127,505 was studied across 13 different countries (Table 1). Ten reports (62.5%) assessed mortality differences using a pre-post design,^31,33,34,36,38–43^ five (25.0%) were non-randomized controlled trials,^28,30,35,37^ one (6.3%) was a retrospective cohort study,^32^ and one (6.3%) used quasi-experimental difference-in-differences methodology.^29^ Eight reports (50.0%) included populations from rural areas,^28,30,35,37^ and 10 reports included populations from urban areas (62.5%);^31,33,35,37–41,43^ Table 1.

**Table 1.** Descriptive Characteristics of Included Reports, Ordered by Intervention Type

Report	Phase	Type	n	RR	Lower	Upper	Design	Location	Cause of Mortality
Murad, et al 2010^28^	Layperson	Trauma Care Training Courses	1,341	0.63	0.44	0.91	Nonrandomized Controlled Trial	Iraq (Rural)	All Trauma
Patel, et al 2016^29^	Layperson	Transport	1,406	0.41	0.15	1.07	Difference-in-Differences	Ghana (Rural)	All Causes
De Costa, et al 2009^30^	Layperson	Transport	10,305	0.43	0.22	0.85	Nonrandomized Controlled Trial	India (Rural)	Maternal Mortality
Mitra, et al 2020^31^	Prehospital	Notification Systems	471	0.94	0.72	1.23	Pre-Post, Unadjusted	India (Urban)	All Trauma
Liberman, et al 2005^32^	Prehospital	Notification Systems	72,073	0.61	0.39	0.94	Observational, Risk-Adjusted	Canada (Province-Wide)	All Trauma
Husum, et al 1999^33^	Prehospital	Trauma Care Training Courses	3,890	0.52	0.45	0.6	Pre-Post, Unadjusted	Afghanistan (Urban)	Combat Trauma
Husum, et al 2003^34^	Prehospital	Trauma Care Training Courses	283	0.45	0.27	0.76	Pre-Post, Unadjusted	Cambodia (Rural), Iraq (Rural)	Landmine Injuries
Saghafinia, et al 2009^35^	Prehospital	Trauma Care Training Courses	471	0.42	0.18	0.99	Nonrandomized Controlled Trial	Iran (Rural and Urban)	Penetrating Injuries
Wisborg, et al 2008^36^	Prehospital	Trauma Care Training Courses	230	0.33	0.17	0.62	Pre-Post, Unadjusted	Iraq (Rural)	Penetrating Injuries
Arreola-Risa, et al 2004* (Monterrey)^37^	Prehospital	Trauma Care Training Courses	866	0.72	0.47	1.11	Nonrandomized Controlled Trial	Mexico (Urban)	All Trauma
Arreola-Risa, et al 2004* (San Pedro)^37^	Prehospital	Trauma Care Training Courses	510	1.09	0.44	2.71	Nonrandomized Controlled Trial	Mexico (Urban)	All Trauma
Arreola-Risa, et al 2007^38^	Prehospital	Trauma Care Training Courses	2,663	0.28	0.12	0.63	Pre-Post, Unadjusted	Mexico (Urban)	All Trauma
Ali, et al 1993^39^	Health Facility	Trauma Care Training Courses	813	0.5	0.43	0.58	Pre-Post, Unadjusted	Trinidad and Tobago (Urban)	All Trauma
Petroze, et al 2015^40^	Health Facility	Trauma Care Training Courses	2,110	0.78	0.58	1.06	Pre-Post, Unadjusted	Rwanda (Urban)	All Trauma
Wang, et al 2010^41^	Health Facility	Trauma Care Training Courses	820	0.82	0.61	1.1	Pre-Post, Unadjusted	China (Urban)	All Trauma
Altamirano, et al 2020^42^	Health Facility	Trauma Systems Organization	28,026	0.78	0.61	0.99	Pre-Post, Unadjusted	Ecuador (Rural)	All Trauma
Hashmi, et al 2013^43^	Health Facility	Trauma Systems Organization	1,227	0.52	0.34	0.8	Pre-Post, Unadjusted	Pakistan (Urban)	All Trauma

Note: *Data disaggregated by site; drawn from same report (Arreola-Risa, et al 2004).

Of the seven intervention subcategories, one report studied layperson trauma care training courses,^28^ two studied layperson transport,^29,30^ two studied prehospital notification systems,^31,32^ seven studied prehospital trauma care training courses,^33–38^ three studied facility-based trauma care training courses (eg, Primary Trauma Care Course, Advanced Trauma Life Support),^39–41^ and two studied facility-based trauma team organization and activation protocols (Table 1).^42,43^

### Pooling Intervention Effect Estimates

For the layperson transport category, a relative risk of 0.42 (95%CI, 0.24-0.74) was observed with no heterogeneity (I^2^ = 0%). For the prehospital notification systems category, a relative risk of 0.79 (95%CI, 0.51-1.19) was observed with substantial heterogeneity (I^2^ = 63% [0%-92%]). The prehospital trauma care training courses category had a relative risk of 0.52 (95%CI, 0.46-0.59) with low-moderate heterogeneity (I^2^ = 36% [0%-73%]), which was relatively similar to facility-based trauma care training courses category (relative risk of 0.67; 95%CI, 0.48-0.92) but with higher heterogeneity (I^2^ = 85% [54%-95%]). Facility-based trauma team organization and activation protocols category had a relative risk of 0.66 (95%CI, 0.45-0.97) with moderate heterogeneity (I^2^ = 61% [0%-91%]).

### Intervention Modeling

**Figure 3.**
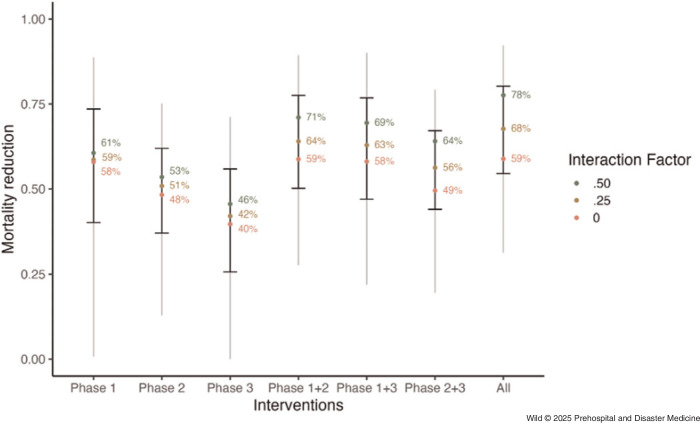
Modeled Estimates of Mortality Reduction per Phase of Emergency Care Intervention. Note: Colored dots indicate median estimates at varying levels of interaction factors, a constant modeling the extent to which aggregated interventions exhibit overlapping effectiveness. Error bars indicate 95% uncertainty intervals, representing the 5th and 95th percentiles of values across all simulations. Grey lines indicate the range between minimum and maximum values across all simulations.

From the primary model, a 68% reduction in mortality was observed when implementing the full set of emergency care interventions in a region with no prior implemented interventions (95%UI, 57%-79%); Table 2 and Figure 3. Although all phases demonstrated significant potential for interventions to reduce mortality, interventions in Phase 1 (layperson first response) had a higher modeled aggregate mortality reduction (59% [95%UI, 40%-74%]) compared to interventions in Phase 2 (prehospital) and Phase 3 (facility-based) - (52% [95%UI, 39%-62%] and 42% [95%UI, 26%-55%], respectively).

**Table 2.** Modeled Estimates of Mortality Reduction by Interaction Factor (IF) and Intervention Phase

Phase	IF 100%	IF 75%	IF 50%	IF 25%	IF 0%
Phase 1	0.73 (0.53-0.85)	0.65 (0.47-0.76)	0.61 (0.43-0.74)	0.59 (0.4-0.74)	0.58 (0.38-0.74)
Phase 2	0.61 (0.44-0.74)	0.58 (0.43-0.7)	0.55 (0.41-0.66)	0.52 (0.39-0.62)	0.5 (0.37-0.6)
Phase 3	0.56 (0.33-0.71)	0.5 (0.31-0.65)	0.46 (0.28-0.59)	0.42 (0.26-0.55)	0.4 (0.24-0.53)
Phase 1+2	0.9 (0.8-0.95)	0.8 (0.69-0.87)	0.71 (0.59-0.81)	0.64 (0.51-0.77)	0.59 (0.46-0.74)
Phase 1+3	0.88 (0.77-0.94)	0.78 (0.65-0.86)	0.69 (0.55-0.8)	0.63 (0.48-0.76)	0.58 (0.42-0.74)
Phase 2+3	0.83 (0.7-0.9)	0.74 (0.62-0.82)	0.65 (0.54-0.74)	0.57 (0.46-0.67)	0.51 (0.4-0.6)
All	0.95 (0.9-0.98)	0.88 (0.8-0.92)	0.78 (0.68-0.86)	0.68 (0.57-0.79)	0.59 (0.47-0.74)

Note: Numbers in parentheses indicate 95% uncertainty intervals, representing the 5th and 95th percentile values across all simulations. Interaction factor refers to a parameter that indicates the extent to which multiple interventions interact to reduce mortality in aggregate.

**Figure 4.**
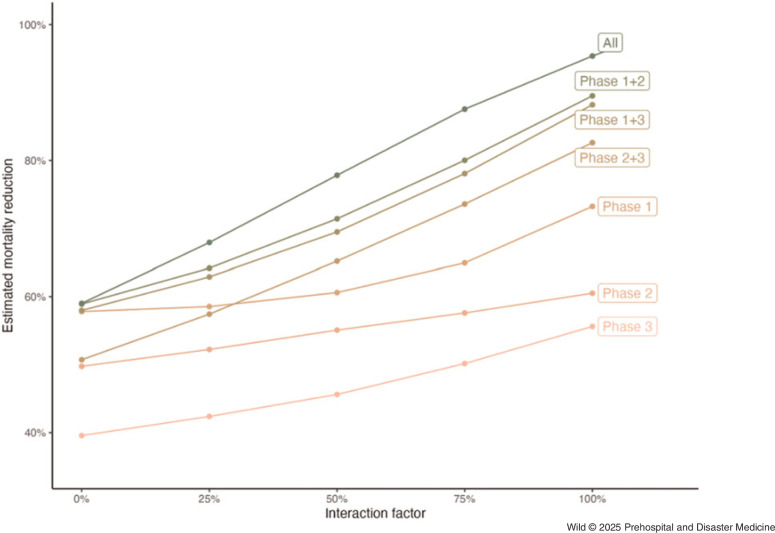
Relationship between Modeled Mortality Reduction and Interaction Factor, Stratified by Intervention Phases. Note: Interaction factor refers to a model parameter that determines the extent to which multiple interventions interact to reduce mortality in aggregate: a 0% interaction factor means that interventions do not overlap and only the most effective single intervention is applied; a 100% interaction factor means that all interventions fully overlap and can be applied sequentially with no diminishing marginal returns.

In an ideal scenario where emergency care interventions are fully independent of each other (ie, the interaction factor is set to 100%, meaning the effects of each intervention are entirely additive), a total mortality reduction of 95% (95%UI, 90%-98%) was observed. In a conservative scenario where emergency care interventions fully share the same mechanisms of action (ie, the interaction factor is set to 0%, meaning additional interventions provide no incremental benefit), a total mortality reduction of 59% (95%UI, 47%-74%) was observed. The estimated mortality reduction increased substantially as the interaction factor increased (Figure 4).

## Discussion

Despite the increasing toll of explosive weaponry on civilian populations, trauma care advances from military and high-income civilian settings have not systematically been translated to improve the care of civilian EO casualties in LRCS.^44^ This study modeled the potential mortality reduction of a coordinated set of emergency care interventions for EO casualties that can be feasibly implemented through strengthened partnership between HMA and health stakeholders at the local, national, and multilateral levels. Even in LRCS where formal emergency care systems are limited, interventions along a continuum of care from point-of-injury to health facility hold potential to reduce preventable death (C-CCC; Supplementary Figure 1). Using the most conservative estimates, implementation of the package of interventions described herein (ie, layperson first-responder trainings, layperson transport, prehospital trauma care trainings, prehospital casualty notification systems, facility-based trauma care trainings, and facility-based trauma team organization) could yield a mortality reduction of up to 59%. This finding is consistent with Joint Trauma System mortality reduction within a military context.^15^

### Policy Context and Implications: Humanitarian Mine Action

While HMA stakeholders are not primarily responsible for providing emergency care to civilian EO casualties, they hold currently under-leveraged capacity to strengthen the quality of emergency care in conflict and post-conflict settings. Several existing policy frameworks exist to structure these obligations and opportunities. First, IMAS 13.10 on Victim Assistance delineates a responsibility to ensure the provision of adequate emergency care to EO victims.^13^ Acknowledging the increasing threat of antipersonnel mines of an improvised nature/IEDs, the 2024 United Nations Secretary-General Report, “Countering the Threat Posed by Improvised Explosive Devices” contained provisions to strengthen emergency care for IED victims: “Immediate post-blast care is vital to reducing preventable deaths and disabilities from improvised explosive devices incidents.”^45^ Additionally, the Antipersonnel Mine Ban Convention 5^th^ Review Conference in Cambodia, November 2024 contains provisions for strengthening emergency care, specifically identifying layperson first-responder trainings as a priority.^46^ To fulfill these responsibilities, a coordinated plan of action that engages local and national authorities from both the health and HMA sectors (eg, locoregional professional societies for surgery and trauma care, national ministries of health, national mine action authorities) as well as multilateral bodies such as the WHO can be developed for step-wise pilots of each intervention contained in this care bundle. Pilot studies should be conducted to generate implementation toolkits (eg, study instruments for needs assessments, curricula, monitoring and evaluation, workshop guides for community engagement, and context-specific adaptation) that can support rapid, system-wide scaling and context-appropriate adaptation in other LRCS. An ideal target would be for adoption of these standards as an appendix to IMAS 13.10 and to the Joint Operational Framework on Health and Protection in order to contextualize these activities within the broader Humanitarian Response System.^47^

### Policy Context and Implications: Emergency Health Care

At the 76^th^ World Health Assembly in 2023, the WHO Resolution “Integrated Emergency, Critical, and Operative (ECO) Care for Universal Health Coverage and Protection from Health Emergencies” (ECO resolution 76.2) was adopted.^48^ The ECO resolution highlights the interdependency of the services and stakeholders required to provide high-quality care for life-threatening conditions regardless of etiology (eg, trauma, post-partum hemorrhage). The priorities of the ECO agenda are reflected in the findings that mortality reduction increased substantially as the interaction factor between phases of care is increased, emphasizing the importance of an integrated approach. The WHO-led ECO initiatives span the entire continuum of care from the community (in the context of EO casualties, point-of-injury) to the health facility level.

Closer engagement with HMA actors can potentiate these initiatives by leveraging HMA capabilities and infrastructure to disseminate and scale WHO ECO interventions such as those contained within the Emergency Care Toolkit.^49^ As one example, at the community level, the WHO’s Community First Aid Responder (CFAR) training seeks to build capacity by strengthening the emergency first aid knowledge of community-based first responders with designated links to the health system to ensure appropriate and timely patient transfer. Similar training for emergency care providers can be strengthened with the Basic Emergency Care (BEC) course and more advanced trauma surgical modules via WHO Academy. Mine action operators conduct large-scale EORE activities in communities affected by the threat of EO. For example, in 2022, EORE activities recorded by the United Nations Mine Action Service (New York USA) alone reached 5,535,800 beneficiaries.^50^ This figure does not include the EORE activities of many other mine action operators (eg, Mines Advisory Group [MAG; Manchester, United Kingdom], The HALO Trust [United Kingdom], Norwegian People’s Aid [Norway], HI, DanChurchAid [Denmark], Foundation Suisse de Déminage [Geneva, Switzerland], Danish Refugee Council [Copenhagen, Denmark], and ICRC). Systematic integration of a conflict-adapted CFAR curriculum adopted as a sector standard for best practices in enhanced EORE could dramatically scale the reach of this intervention bundle in conflict- and post-conflict communities globally.

In addition to leveraging EORE activities and de-mining medics for community, prehospital, and facility-based trauma care trainings, numerous other opportunities exist for engagement between HMA and emergency care systems strengthening, as have been detailed in previous reports.^18,19^ As just one example, mine action operators currently conduct trauma care capability assessments at health facilities to inform casualty evacuation procedures for their operations. Such activities could be conducted in collaboration with local health authorities using a standardized instrument such as the Index for Trauma Capacity in Low-Income Countries (INTACT) tool to create casualty notification systems as well as benchmark improvements in infrastructure and trauma care capabilities over time.

### Paths Forward

Cooperative action between HMA and health stakeholders to conduct stepwise implementation of the interventions included in analysis requires engagement from a diverse nexus of actors. Necessary stakeholders within the HMA sector include national mine action authorities, HMA operators, and international governing bodies such as the Anti-Personnel Mine Ban Convention Implementation Support Unit (ISU) and the Convention on Cluster Munition ISU. Within the health sector, relevant actors include everything from community health workers to prehospital personnel (where such structures exist), national and regional surgical societies, ministries of health, and the WHO (specifically, the Clinical Services and Systems Unit, Emergency Medical Teams Initiative). Building off of the Joint Operational Framework on Health and Protection, clear operational guidance for coordination between HMA and health actors must be delineated to ensure that intervention delivery as well as monitoring, evaluation, and reporting are conducted in a standardized manner. The foundation for such sector-wide coordination was first laid during a plenary session at the 26^th^ United Nations International Meeting of Mine Action National Directors in Geneva, Switzerland, June 2023.^14^ While a repository of open-access toolkits will be developed (supplemental to WHO resources such as the WHO Emergency Care Toolkit), context-appropriate adaptation is critical, and this process must directly engage communities and emergency care providers in EO-affected settings.^51^ The first such collaborative pilot is currently taking place in the Sahel, where MAG and the Explosive Weapons Trauma Care Collective (EXTRACCT) are working with local partners to conduct joint delivery of EORE with conflict-adapted CFAR training among communities affected by IEDs in Burkina Faso (West Africa).^44^

## Limitations

This study had several limitations, primarily related to the quality and quantity of prior reports on emergency care interventions. First, due to the nature of evaluating emergency care interventions in low-resource environments, the reports leveraged in this study did not employ randomized controlled trials and instead used approaches like non-randomized controlled trials, pre-post designs, and quasi-experimental methodology. The results from existing reports are potentially subject to unmeasured confounding. Secondly, some intervention subcategories had small numbers of reports for their respective meta-analyses, yielding heterogeneity and uncertainty as to the true pooled effect size. These factors were mitigated by leveraging uncertainty quantification via Monte Carlo simulations, but only enhanced primary data collection will improve precision of measured intervention efficacy. Thirdly, most reports analyzed did not provide cause-specific mortality, preventing the modeling of cause-specific mortality pathways and instead aggregating all mortality-reduction interventions together. Such an approach may over-estimate aggregated mortality reduction as different interventions may act on the same cause-specific mortality pathways. Adjustments were made for this by introducing an interaction factor to diminish estimates of aggregate mortality reduction, thereby allowing the provision of upper and lower bounds of total mortality reduction, but the true interaction factor remains unknown and a source of uncertainty – these results should therefore be interpreted in the context of their full uncertainty intervals instead of singular point estimates. Additionally, the approach of using a single parameter to model interactions between interventions may not fully reflect heterogeneity in how different interventions may interact – future work should investigate and validate the efficacy of combined interventions. Further, these modeled estimates of mortality reduction will be subject to significant variability in real-world scenarios, and patient data to corroborate these estimates are frequently challenging to obtain in LRCS. Finally, the generalizability of these findings will be highly context-specific depending on resource availability in any given setting. Despite these limitations, these findings are consistent with relative risk reductions from capacity building initiatives reported by the United States Military Joint Trauma System and allow reasonable conclusions to be drawn about opportunities to reduce EO-related mortality among civilians with improved coordination between HMA and local emergency care systems.

## Conclusion

Numerous interventions with potential applicability to EO casualties have been demonstrated to reduce trauma-related mortality in both high- and low-resource trauma systems. Despite evidence of this mortality reduction potential, such interventions have not yet systematically reached local EO casualties in LRCS. Enhanced coordination between HMA and health actors to implement a structured set of emergency care interventions holds potential to significantly reduce preventable death among civilians affected by conflict.

## Supporting information

Wild et al. supplementary material 1 Wild et al. supplementary material

Wild et al. supplementary material 2 Wild et al. supplementary material

## Supplementary Material

To view supplementary material for this article, please visit https://doi.org/10.1017/S1049023X25101283
