# Supplementary material for: Emergency Care Interventions for Victims of Explosive Ordnance Reduce Mortality: A Modeling Study
Source: Prehosp Disaster Med. 2025 Aug 20;40(4):204–13. doi: 10.1017/S1049023X25101283 (PMC12809202; doi:10.1017/S1049023X25101283)
Supplement: Wild et al. supplementary material 2 — Wild et al. supplementary material [file S1049023X25101283sup002.docx]

## **Supplementary Information**

**Supplementary Figure 1: Civilian casualty care chain (C-CCC)**

**Supplementary Table 1:** **Heterogeneity estimates from meta-analyses pooling effect estimates within emergency care intervention categories**

**Supplementary Figure 2: A leave-one-out meta-analysis for studies in the Prehospital Trauma Care Training Courses subcategory to assess for outlier studies**

**Supplementary Figure 3: A leave-one-out meta-analysis for studies in the Health Facility Trauma Care Training Courses subcategory to assess for outlier studies**

**Supplementary Figure 1. Civilian casualty care chain (C-CCC)^1^**

**
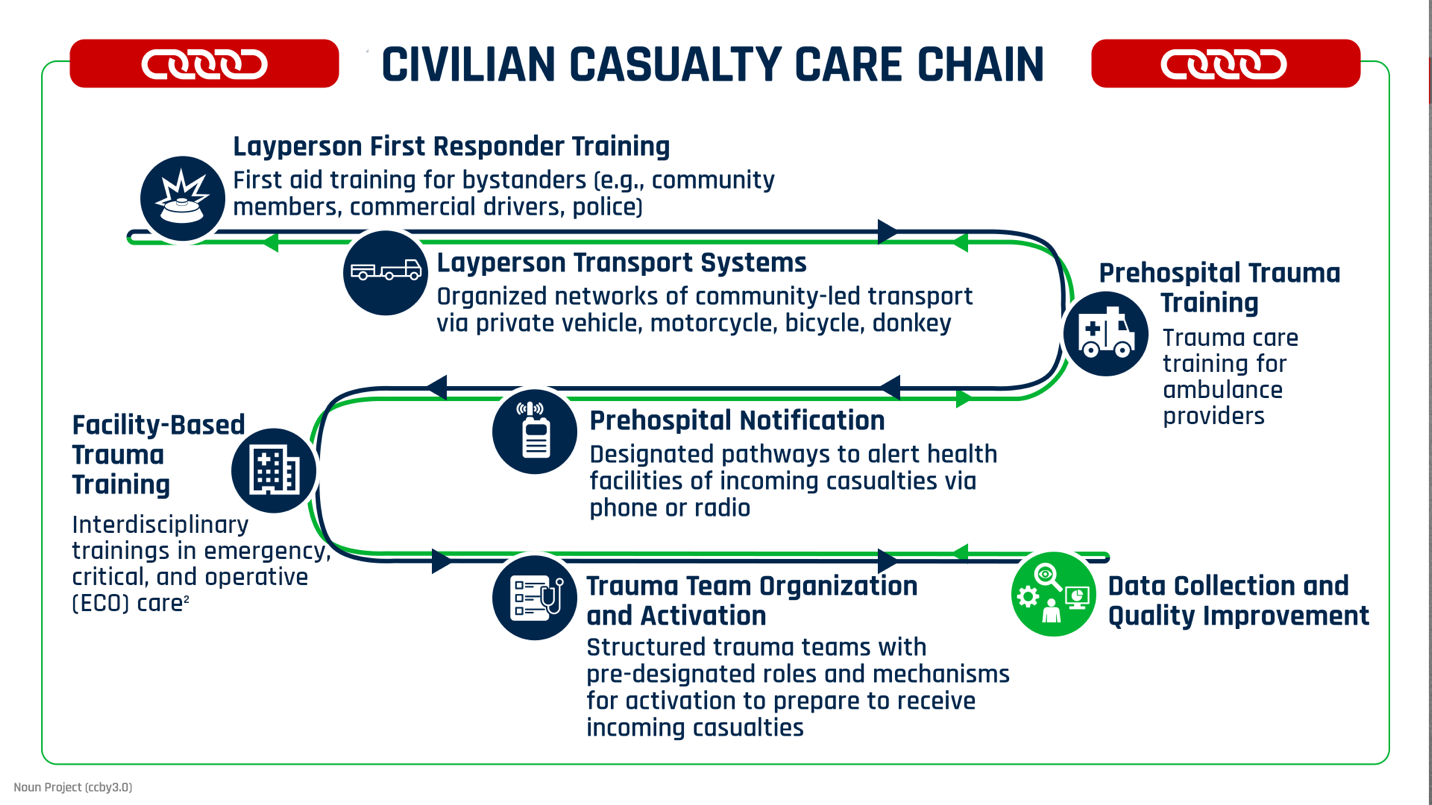
**

1. The C-CCC outlines a selected set of interventions that represent opportunities for HMA stakeholders to engage in health sector initiatives to improve emergency care of EW casualties. The C-CCC is not itself an emergency care pathway, as it lacks many of the emergency care system components needed for a continuum of response. Rather, it highlights specific areas of targeted action in which HMA stakeholders might leverage their existing capabilities, infrastructure, and operations to support local emergency care systems to improve trauma care for EW casualties.

2. Interdisciplinary refers to the interprofessional nature of robust emergency, critical, and operative care, engaging all relevant health care providers including physicians, surgeons, nurses, and health officers.

**Supplementary Table 1:** **Heterogeneity estimates from meta-analyses pooling effect estimates within emergency care intervention categories^1^**

| **Intervention category** | **I^2^ (95% CI)** | **T^2^ (95% CI)** | **H (95% CI)** | **Q** | **Q p-value** |
| --- | --- | --- | --- | --- | --- |
| Layperson Transport | 0 (NA) | 0 (NA) | 1 (NA) | 0.01 | 0.9376 |
| Prehospital Notification Systems | 0.63 (0-0.92) | 0.059 (NA) | 1.65 (1-3.43) | 2.71 | 0.0998 |
| Prehospital Trauma Care Training Courses | 0.36 (0-0.73) | 0.063 (0-0.8743) | 1.25 (1-1.92) | 9.33 | 0.1559 |
| Health Facility Trauma Care Training Courses | 0.85 (0.54-0.95) | 0.0647 (0.009-3.0086) | 2.55 (1.48-4.39) | 12.98 | 0.0015 |
| Health Facility Organization & Activation Protocols | 0.61 (0-0.91) | 0.0482 (NA) | 1.6 (1-3.33) | 2.57 | 0.1086 |

## 1. All intervals presented are 95% confidence intervals. NA indicates incalculable heterogeneity confidence intervals in cases of low sample size or near-zero heterogeneity.

## **Supplementary Figure 2: A leave-one-out meta-analysis for studies in the Prehospital Trauma Care Training Courses subcategory to assess for outlier studies**

**
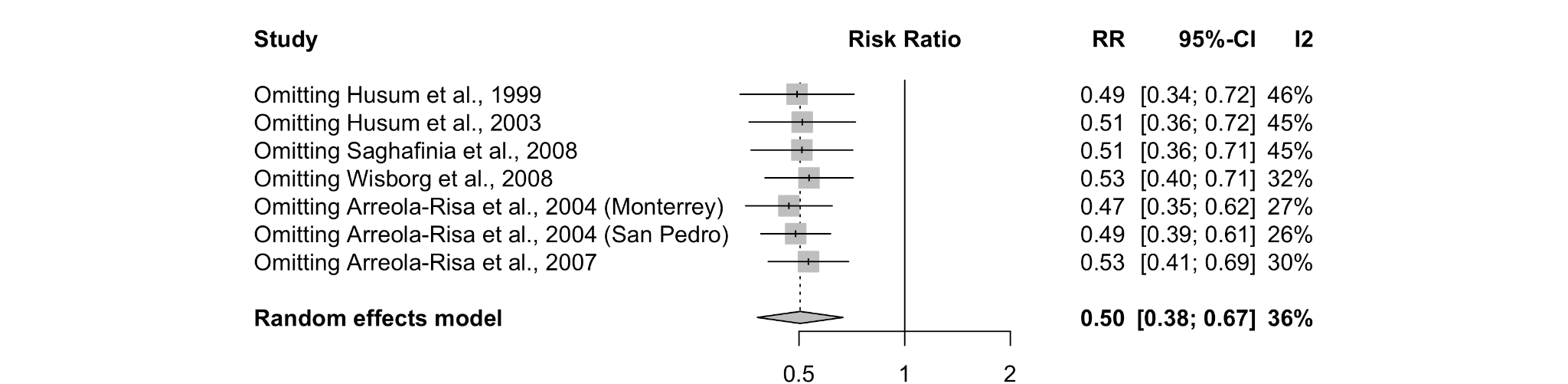
**

## **Supplementary Figure 3: A leave-one-out meta-analysis for studies in the Health Facility Trauma Care Training Courses subcategory to assess for outlier studies**

**
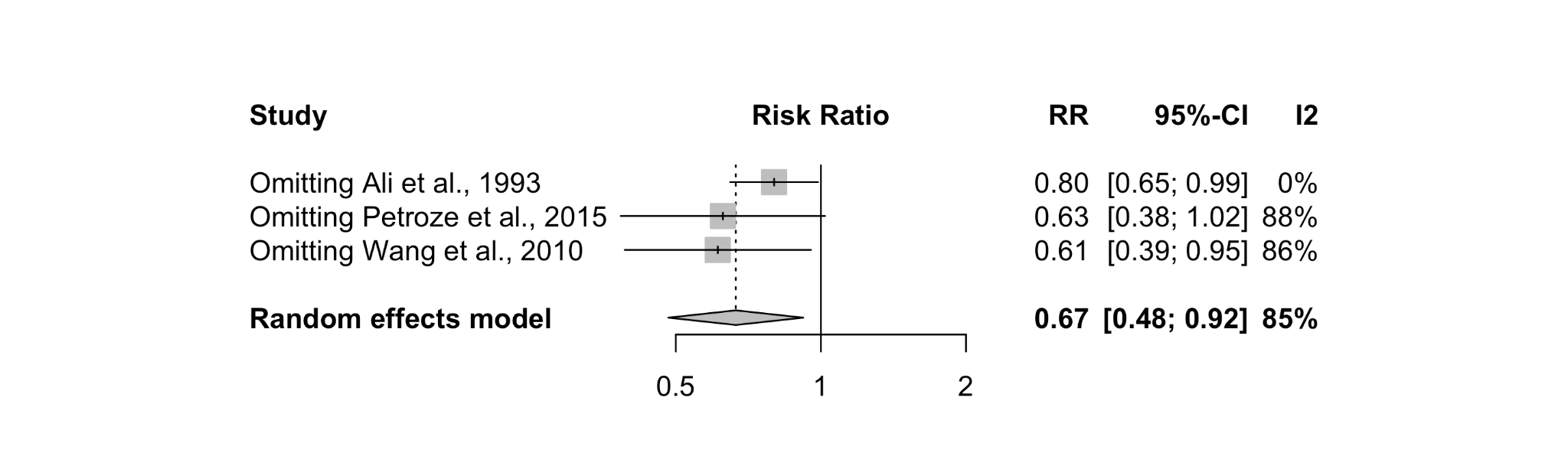
**
